# Argon plasma coagulation with atezolizumab and chemoradiation in lung pleomorphic cancer showed a remarkable response: Case report

**DOI:** 10.1097/MD.0000000000026149

**Published:** 2021-05-28

**Authors:** Masanori Harada, Keisuke Morikawa, Namio Kagoo, Yutaro Ito, Tsutomu Kubota, Koshiro Ichijo, Eisuke Mochizuki, Masahiro Uehara, Shun Matsuura, Kazuyo Yasuda, Masaru Tsukui, Naoki Koshimizu

**Affiliations:** a Department of respiratory medicine; b Department of pathology, Fujieda Municipal General Hospital, 4-1-11 Surugadai, Fujieda, Japan.

**Keywords:** chemoradiotherapy, immune checkpoint inhibitor, lung pleomorphic carcinoma, radiofrequency, synergistic effect

## Abstract

**Rationale:**

Lung pleomorphic carcinoma (LPC) is generally resistant to chemotherapy or radiotherapy. However, a combination of immune checkpoint inhibitors and radiotherapy has a remarkable efficacy against LPC.

**Patient Concerns and Diagnoses:**

Here, we report the case of a 50-year old man diagnosed with progressive LPC. The tumor invaded the carina and predominantly obstructed the right main bronchus; therefore, a combination of palliative chemoradiotherapy and atezolizumab was initiated. However the trachea was gradually obstructed.

**Intervention and Outcome:**

Argon plasma coagulation (APC) was performed to prevent tumor invasion. After three APC sessions, the tumor showed a necrotic change and was easily excised using biopsy forceps.

**Lessons:**

A combination of chemoradiotherapy, atezolizumab, and APC showed a good efficacy, and the patient had a good response to atezolizumab maintenance therapy. Multidisciplinary treatments, such as a combination of immune checkpoint inhibitors and APC, could have synergistic efficacy in lung cancer.

## 1 Introduction

Treatment with immune checkpoint inhibitors is very effective for non-small cell lung cancer (NSCLC). Using a combination of atezolizumab and chemotherapy as first-line treatment has improved the progression-free survival of patients with NSCLC, regardless of the programmed death-ligand 1 (PD-L1) expression level.^[1]^ A combination of chemoradiotherapy and durvalumab, an anti-PD-L1 inhibitor, is highly efficacious,^[2]^ suggesting that both radiation and chemotherapy have a synergistic effect with PD-L1 inhibitors in NSCLC. Lung pleomorphic carcinoma (LPC), a rare type of poorly differentiated NSCLC, is generally resistant to chemotherapy or radiotherapy^[3]^; however, some studies have reported a marked response of LPCs to nivolumab with or without local radiotherapy.^[4–8]^ Herein, we reported a rare case of progressive LPC that improved markedly after treatment with a combination of argon plasma coagulation (APC), chemoradiotherapy, and atezolizumab.

## 2 Case report

**Figure 1:**
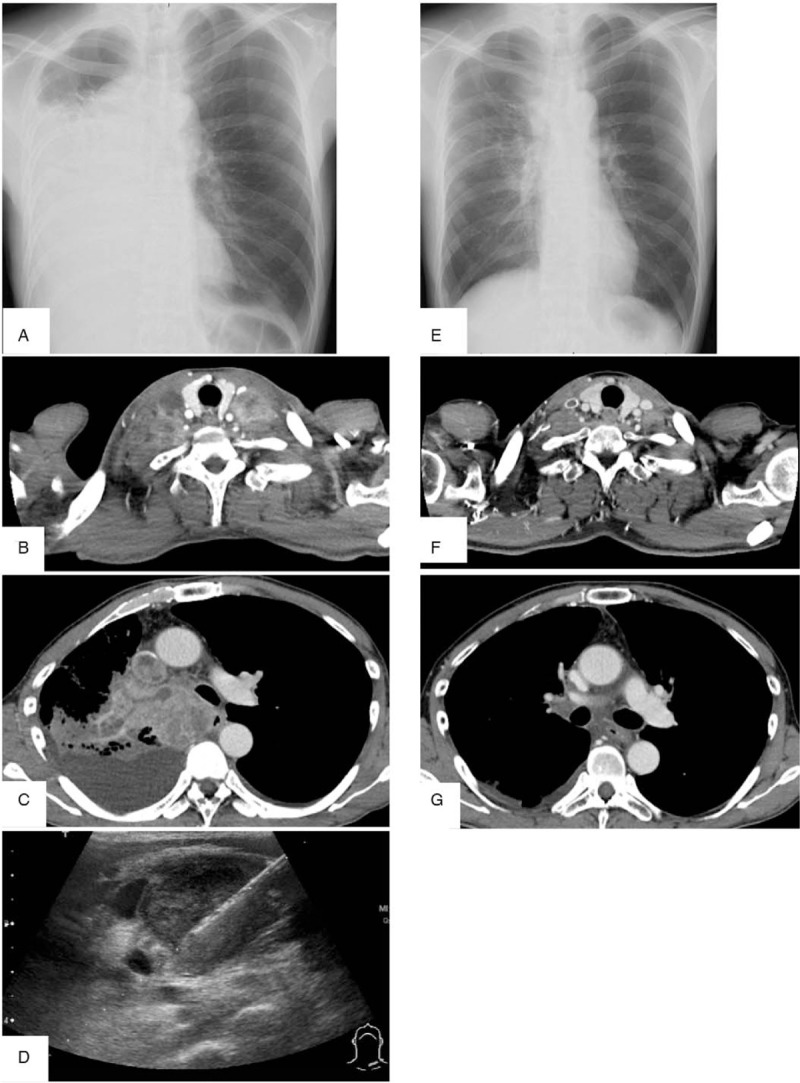
Chest radiography and computed tomography findings. A-C: before treatment initiation, E-G: after two cycles of combination therapy, D: percutaneous ultrasound appearance of needle biopsy of the left side of a cervical lymph node.

**Figure 2:**
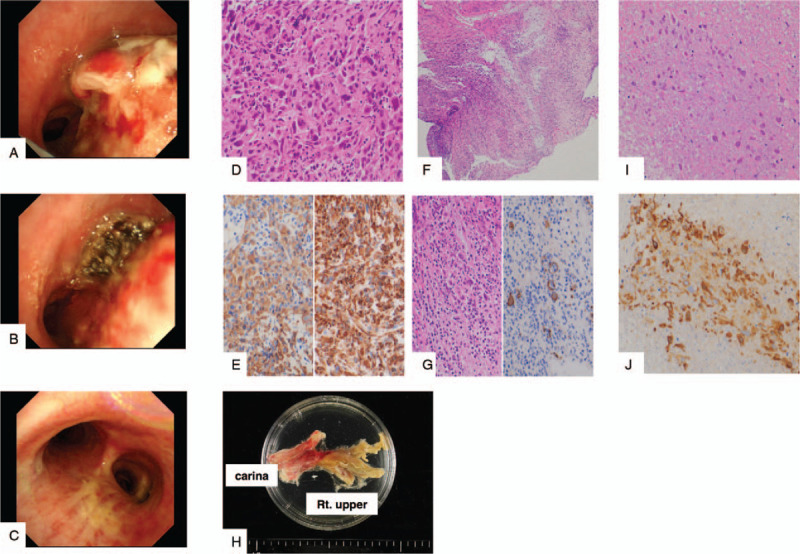
A-C: endobronchial appearances, A: Treatment initiation, B: after several argon plasma coagulation treatments, C: after two cycles of combination therapy, D: Histological examination of left cervical lymph node showing proliferation with spindle and giant tumor cells only (Hematoxylin and Eosin stain, x400 magnification). E: Tumor cells stained positive on immunohistochemistry for CK AE1/AE3 and vimentin (left, CK AE1/AE3, x400 magnification; right, Vimentin, x400 magnification) F: Necrosis and massive neutrophil infiltration in the endobronchial tumor of the carina (Hematoxylin and Eosin stain, x100 magnification). G: The other tumor cells are necrotic and stain positively for CK AE1/AE3 (left; Hematoxylin and Eosin stain, x400 magnification; right, CK AE1/AE3, x400 magnification) H: Macroscopic appearance of the endobronchial tumor molding the bronchial form. I: The excised mass contains sparse degenerated residual cancer cells in the necrotic tissue (Hematoxylin and Eosin stain, x400 magnification), J: Residual tumor cells stain positively for CK AE1/AE3 (CK AE1/AE3, x400 magnification).

A 50-year-old male agricultural worker consulted his primary care physician with dyspnea, cough, and fever, which had started one month before this consultation. He was a current smoker with no significant past or family history. His body temperature was 37.3°C, and his oxygen saturation was 92% in room air. Chest radiography and computed tomography revealed a right pleural effusion and large tumor that was dominant in his right main bronchus. There were multiple enlarged mediastinal, right hilar, and bilateral cervical lymph nodes. There were multiple small nodes in his left lung (Fig. 1A-C). Metastases were detected in his liver and left adrenal gland. Intravascular thrombi were found in the superior vena cava and right subclavian vein (Fig. 1B, C). His serum tumor marker levels were slightly elevated (neuron-specific enolase, 18.2 ng/mL; cytokeratin fragment, 6.3 ng/mL), and his D-dimer titer was high (14.7 μg/mL). However, other serum workup results were normal. Thoracoscopic pleural biopsy and ultrasound-guided needle biopsy of the left cervical lymph node (Fig. 1D) were performed. A microscopic examination of the pleural mass and cervical lymph node lesions revealed tumor proliferation of only spindle and giant cells (Fig. 2D). On immunohistochemical examination, the tumor cells were positive for CK AE1/AE3, vimentin (Fig. 2E), and CK7; weakly positive for TTF1 and CK5/6; and negative for CK20, p40, WT-1, and calretinin. Histopathological examination of the biopsies suggested LPC. Based on these results, the patient was diagnosed with progressive cT4N3M1c stage IVB LPC. There were no mutations of oncogenic driver genes (epidermal growth factor receptor, ROS1, and BRAF); however, the percentage of PD-L1-positive cells was 100%. A combination of atezolizumab (1200 mg), carboplatin (AUC6), and nab-paclitaxel (100 mg/m^2^) chemotherapy was administered. Apixaban (10 mg), an anticoagulant, was initiated. The tumor was invading areas superior to the carina, which were likely to get totally obstructed; therefore, 30 gray palliative radiation therapy of the mediastinal lesion was initiated simultaneously. A sudden onset of dyspnea and desaturation were observed one week after treatment initiation. Obstruction of the trachea by the tumor was suspected, and bronchoscopy was performed. The tumor had invaded the left bronchi (Fig. 2A); therefore, the tumor was excised with biopsy forceps, and additional APC therapy with 30 watts of electronic energy was administered to relieve symptoms and prevent further tumor invasion. Although no change was observed in the first two weeks after atezolizumab initiation (Fig. 2B), histological examination of the excised lung fraction revealed necrosis of the tumor cells (Fig. 2F, G). After two cycles of combination therapy, the invading tumor could be excised easily using biopsy forceps (Fig. 2C). Macroscopic examination of the excised tumor tissue showed severe necrosis (Fig. 2H). Histological examination showed sparse degenerated residual tumor cells in the abundant necrotic tissue, indicating a marked therapeutic effect (Fig. 2I, J). The patient has maintained a good response to triweekly atezolizumab therapy after four treatment cycles (Fig. 1E-G). Moreover, he achieved near-complete remission of the LPC without any recurrence, even after he was followed up for one year.

## 3 Discussion

Our patient showed a remarkable response to APC with atezolizumab and chemoradiotherapy. LPC is a rare phenotype of NSCLC and is generally resistant to chemoradiotherapy. Pathologically, LPC is defined as a poorly differentiated NSCLC, with at least 10% spindle and/or giant cells or a carcinoma consisting only of spindle and giant cells. LPC constitutes only about 0.1% to 0.4% of all malignant tumors; therefore, it has not been evaluated clinically fully.^[3]^ Recently, another anti-PD-L1 inhibitor, durvalumab, was proven highly effective after concurrent chemoradiotherapy in patients with stage III NSCLC.^[2]^ Radiotherapy and chemotherapy do not only promote PD-L1 expression in tumors, but also the abscopal effect against tumor antigens.^[9]^ Our patient showed a high PD-L1 expression in cancer cells; therefore, this could explain the patient's good response to the combination of ICI and chemoradiotherapy.

A previously reported case of progressive NSCLC showed a very good response to ICI in combination with radiofrequency ablation (RFA) and adjuvant atezolizumab.^[10]^ RFA waves are converted into heat to achieve local temperatures capable of inducing tissue destruction.^[11]^ Traditionally, argon plasma coagulation (APC) is the primary endoscopic modality for the treatment, and RFA is an alternative to APC due to its larger per-treatment surface area or reproducible depth of treatment.^[12]^ These methods could induce an antigen source to trigger an anti-tumor immune response. Treatment with RFA resulted in enhanced systemic antitumor T-cell immune responses and tumor regression.^[13]^ Liangrong et al. studied the RFA-induced immune responses in tumor tissues from patients with cancer. In this report, RFA initially enhanced a strong T-cell-mediated immune response in the tumor, and the tumor quickly overcame the immune responses by inhibiting the function of CD8+ and CD4+ T cells, driving a shift to higher regulatory T-cells and upregulating PD-L1/PD-1 expression. Furthermore, PD-1 inhibitors enhanced T-cell immune responses significantly, resulting in stronger antitumor immunity and prolonged survival.^[14]^ RFA was performed after the administration of immunotherapy for 7 days; this induced rapid tumor growth suppression. Therefore, a combination of RFA and ablation enhanced antitumor efficacy and contributed to improving survival.^[15]^

In our patient, one of the high-frequency ablation methods (multiple APC therapy sessions) was used with chemoradiotherapy and ICI therapy, and the LPC was completely excised from the lesion. In the palliative setting of central airway obstruction alleviation, APC is one of the techniques that provides immediate relief. The mechanism of APC is similar to that of RFA, which involves the conversion of to heat tissue. In the noncontact mode using an argon plasma jet, APC also clears the pool of mucus and blood and conducts electrons around the corner.^[16]^ APC is not only a safety procedure in clinical settings but is also an inducer of anti-tumor immune responses like RFA. Furthermore, in a previous case of an unresectable lung tumor with a high PD-L1 expression (tumor proportion score - 90%), a 30 gray palliative irradiation was performed, which was suggested to have an abscopal effect; the same was observed in our patient.^[9]^ Although there are no clinical studies on the antitumor effects of APC, studies on a combination of APC and various immune modulators are required.

## 4 Conclusion

In conclusion, our case showed that a combination of APC and immune checkpoint inhibitors has a high efficacy against LPC. To date, the synergistic mechanisms between APC and PD-L1 blockade remain unclear; however, multidisciplinary treatments, such as palliative irradiation therapy, chemoradiotherapy, ICI, and APC, could have a synergistic effect in lung cancer.

## Acknowledgments

The authors would like to specially thank the staff of the Department of pathology and endoscopy.

## Author contributions

**Conceptualization:** Masanori Harada, Keisuke Morikawa, Namio Kagoo, Yutaro Ito, Tsutomu Kubota, Koshiro Ichijo, Eisuke Mochizuki, Masahiro Uehara, Shun Matsuura, Kazuyo Yasuda, Masaru Tsukui, Naoki Koshimizu.

**Data curation:** Masanori Harada, Keisuke Morikawa, Namio Kagoo, Yutaro Ito, Tsutomu Kubota, Koshiro Ichijo, Eisuke Mochizuki, Masahiro Uehara, Shun Matsuura, Kazuyo Yasuda, Masaru Tsukui, Naoki Koshimizu.

**Formal analysis:** Masanori Harada.

**Investigation:** Masanori Harada.

**Methodology:** Masanori Harada.

**Supervision:** Kazuyo Yasuda, Masaru Tsukui, Naoki Koshimizu.

**Visualization:** Masanori Harada.

**Writing – original draft:** Masanori Harada, Kazuyo Yasuda, Naoki Koshimizu.

**Writing – review & editing:** Masanori Harada, Naoki Koshimizu.
